# Real-Time Forecasting from Wearable-Monitored Heart Rate Data Through Autoregressive Models

**DOI:** 10.1007/s41666-025-00191-y

**Published:** 2025-03-07

**Authors:** Giulio De Sabbata, Giovanni Simonini

**Affiliations:** University of Modena and Reggio Emilia, Modena, Italy

**Keywords:** Heart rate, Forecasting, Wearable sensors, Real-time, Random walk

## Abstract

Heart rate (HR) analysis is of paramount importance in healthcare, particularly for monitoring cardiovascular health, a global concern. The advent of wearable sensors has enabled continuous HR monitoring, with researchers attempting to develop early detection systems by forecasting HR in a univariate fashion. This study analyzes real-world HR time series gathered during participants daily routines to critically assess the predictive power of past HR data in short-term, univariate forecasting. The literature emphasizes a minute-by-minute, univariate forecasting approach, where state-of-the-art predictive models predominantly employ autoregressive integrated moving average (ARIMA). Yet, its superiority has been proved without studying its optimized hyper-parameters, which could not only improve forecast accuracy but also provide valuable insights. By leveraging the interpretability of ARIMA, we tune its hyper-parameters within a minute-by-minute forecasting structure to address the central research question: how does historical HR data contribute to generate accurate short-term HR forecasts? Our analysis finds that the random walk model, a special case of ARIMA, consistently performs comparably to, or even better than, more complex ARIMA specifications. This indicates that HR values alone offer limited predictive power for short-term forecasting, casting doubt on the value of further refinement in univariate models for alarm system development. These findings highlight the limitations of univariate HR forecasting in real-time health monitoring. Rather than increasing model complexity, future research might benefit from exploring alternative approaches to improve early warning system capabilities in real-world settings.

**Supplementary Information:**

The online version contains supplementary material available at 10.1007/s41666-025-00191-y.

## Introduction

The digitization of healthcare has transformed the sector, offering researchers a wealth of new disruptive opportunities. Among these, the proliferation of wearable sensors has generated a huge amount of electronic health data and, thus, fostered further research on one of the major vital signs, the heart rate (HR). HR analysis is crucial in healthcare, especially for the study of cardiovascular diseases, a major global health concern that demands particular attention. Indeed, these diseases continue to be a leading cause of mortality worldwide [1]. Beyond the sheer volume of data, wearable sensors have facilitated real-time monitoring of various physiological parameters in patients throughout their daily routines. In practical healthcare settings, monitoring and alarm systems must operate in real-time, ensuring timely interventions when necessary [2]. The wealth of new studies collecting HR time series has encouraged researchers to elicit insights from this new source of information to develop early detection system [3–10]. Among these, the prevailing approach in recent HR works has leaned towards univariate forecasting of HR values, employing a short-term forecasting strategy with a horizon of 1 min.

Under the assumption that accurate prediction is sufficient for developing effective alarm systems, numerous machine learning and complex deep learning models have been tested to generate real-time forecasts. However, this approach represents a considerable leap, as the efficacy of these systems remains unproven. A primary obstacle remains the scarcity of labeled data in longitudinal studies. This limitation stems from the nature of the available datasets. Studies typically collect data either from healthy individuals or from patients already diagnosed with cardiovascular conditions. Consequently, capturing the precise onset of cardiac events remains elusive. Moreover, the field progress appears to be driven more by comparative analyses of algorithm superiority rather than in view of early detection systems. This focus on algorithmic competition may yield limited benefits in terms of clinical applications.

Given these challenges, we propose that a critical starting point for advancing the field is to assess the performance of univariate forecasting models. This leads us to a central research question: to what extent do predictive models leverage past information in univariate HR forecasting? To address this question, we aim at identifying the most performing specification of autoregressive integrated moving average (ARIMA), which is a highly interpretable model that has shown promising performance compared to other predictive models [5, 6]. By tuning ARIMA hyper-parameters, a rigorous evaluation of model specifications allows us to examine the predictive power of past HR data and assess the potential of univariate models in capturing meaningful patterns. Notably, finance literature emphasizes the role of the random walk model, represented by ARIMA (0,1,0), using it as a benchmark [11]. This derives from the nature of the random walk model, where forecasts are generated by taking a random step away from the previous value, meaning that such a model does not retrieve information from lagged and error term. To assess how past information is leveraged to predict future values, performance of other specifications are compared to that of a random walk model. If the random walk model provides a competitive, if not superior, forecasting baseline, it could be concluded that past information is minimally leveraged. Our findings may help clarify the limitations and potential of univariate HR forecasting, guiding future research towards more effective approaches in cardiovascular health monitoring. Once ARIMA specification is tuned, analysis of residuals is performed to deepen our understanding of model behavior.

Identifying the best specification of hyper-parameters drastically reduces the complexity time of algorithm deployment. Time constraints should be considered when deploying real-time solutions [12, 13]. Further tests are necessary to assess the possibility of deploying the solution in production. While these tests are not covered in this paper, it should be noted that our methodology designed a solution tailored to real-time scenarios. The main contributions of the paper can be summarized as follows:

- Established a predominant forecasting approach. Outlined key passages for designing a forecasting structure in the HR domain, enhancing comparability and comprehension among researchers. Notions of granularity, adaptive learning, and forecasting strategy are crucial for the definition of the forecasting structure;
- ARIMA hyper-parameters testing. This study is the first to conduct a comprehensive testing of different specifications of ARIMA for HR forecasting. Hyper-parameter tuning provides valuable insights into the forecast accuracy associated to historical HR values;
- Assessment of predictive performance in univariate forecasting. We evaluate how historical information is leveraged to generate short-term forecasts in HR univariate setting;
- Implementation of residual diagnostics in the random walk model. Performance of the random walk model is evaluated through residual diagnostics, which represents a means of studying model behavior.

The remainder of the paper is organized as follows: Section 2 deals with the methodology followed to design our minute-by-minute forecasting structure. In particular, a description of the datasets is provided underscoring its adequacy for our purpose. Subsequently, data pre-processing phase is debated focusing on the organization of the dataset and related cleaning tasks. The definition of adaptive learning strategies is further inspected. Lastly, ARIMA algorithm, validation process, and evaluation metrics are analyzed in this section. Section 3 presents results of the tests with a description of the experimental protocol and of the relative residual diagnostics. Section 4 summarizes the findings and relative interpretations. Section 5 discusses the literature, especially in the context of HR and time series forecasting. Section 6 reflects on the possible impact on further research in the field.

## Methods

### Real-World Data: Participants Engaged in Everyday Routines

For this study, we employ two longitudinal datasets, each providing continuous 24-h data gathered through wearable devices. Using wearable sensors to collect data allows to capture a wide range of HR patterns, thereby mirroring real-world variability. Both datasets consist of healthy individuals engaged in their routine daily activities, confirming comprehensive representation of HR dynamics in natural settings.

The first dataset, referred to as MMASH (Multi-Modal Ambulatory Stress and Heart Rate), is titled *A Public Dataset of 24-h Multi-Levels Psycho-Physiological Responses in Young Healthy Adults* [14]. It contains 24 h of continuous psycho-physiological data, e.g., inter-beat interval data, HR data, wrist accelerometry data, sleep quality index, and physical activity. The 22 participants are young healthy males that are monitored during their normal routines.

The second dataset, named *RR interval time series from healthy subjects* (RRITS), encompasses 24-h Holter monitoring data from 147 individuals [15]. The dataset is nearly gender-balanced and includes participants aged 0 to 55, though skewed towards those under 1 year old. While subject-specific factors such as age and gender may affect outcomes in univariate analyses, the inclusion of this heterogeneous sample leads to findings that are more generalizable and less influenced by individual demographic factors.

### Pre-Processing in Heart Rate Time Series

In both datasets, a wearable device is employed to collect data that, thus, may be affected by motion artifacts. Unlike conventional instruments commonly used in clinical settings for HR monitoring, wearable sensors are more prone to generating inaccuracies and errors in the measurements. In MMASH and RRITS, HR data is collected using a PolarH7 sensor and a Holter monitor, respectively. Both measurement systems have been demonstrated to provide sufficient data quality [16, 17]. Notably, the RRITS dataset has been pre-processed and cleaned through the application of a set of criteria defining Holter record validity. In contrast, the authors of the MMASH dataset have not applied any pre-processing techniques to clean the data in their case.

**Fig. 1:**
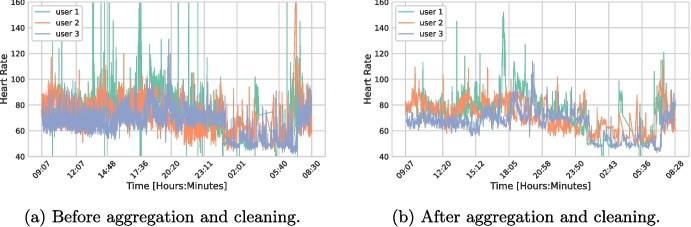
Figures of heart rate time series of 3 random users before and after pre-processing tasks. Before cleaning, several inconsistencies are present which may be caused by motion artifacts. Aggregating helps to smooth heart rate levels whose range was in a non-reliable interval

To ensure data integrity and reliability, the raw individual beats undergo meticulous cleaning, following established protocols in the literature [18]. This process implies evaluating non-physiological HR shifts over time to identify and rectify erroneous measurements. Subsequently, beats are aggregated to obtain a more stable granularity. This approach of capturing the average behavior eliminates extreme values and helps to focus on the general trend. A minute-by-minute structure is designed, disregarding data behavior within shorter time frames (less than a minute). The visual inspection of HR processes before and after aggregation and cleaning, as displayed in Fig. 1 for three random users, demonstrates that these transformations result in smoother time series with reduced abrupt changes.

In the RRITS dataset, there are no indications about the specific time of day when data was gathered. Visual inspection suggests that the authors may not have accounted for time spans during which patients were not wearing the Holter monitor. Conversely, the MMASH dataset provides precise time-of-day information, and the percentages of missing values dataset are generally low, especially after aggregation. Specifically, the majority of users consistently wore the sensor, resulting in only a 1% occurrence of missing values. Even in extreme cases, where missing values reached up to 10% over the course of a full day, this level of data completeness could be deemed acceptable. For such a relatively minor issue, we apply a linear interpolation as an imputation process.

The final pre-processing phase considers stationarity. A process is said to be stationary if all the moments are independent of time series length [19]. Generally, differentiation should be applied to non-stationary time series before processing. Yet, this does not represent a problem since ARIMA can directly handle it. The exploratory phase suggests that the majority of the processes are considered stationary according to the traditional augmented Dickey-Fuller test. To summarize, the limited occurrence of missing values and erroneous measurements underscores the overall high quality of the dataset, offering a solid foundation for our subsequent analysis.

### Adaptive Forecasting Structure

**Fig. 2:**
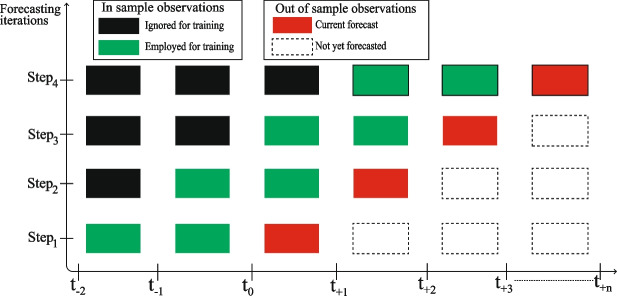
Schema illustrating the updating procedure. This figure clarifies the data workflow, showing how new observations (green), as they become available, are incorporated into the training process. A rolling window mechanism with a fixed size is employed. At each step, the model forecasts the next observation (red) using a one-step-ahead forecasting mechanism. Black cells represent observations ignored during training, while blank cells are observations that will be forecast in future steps

Real-time processing is a critical requirement for modern applications, particularly in fields such as wearable sensor data analysis [20]. These systems must be capable of analyzing continuous data streams as they are generated. This paradigm shift from static to streaming data analysis reflects the growing need for real-world, real-time processing solutions. To address these challenges, the literature highlights adaptive learning strategies for managing real-time data streams, especially in modeling short-term dependencies in HR process. This approach provides effective strategies for handling concept drift, which consists of a gradual shift in data distribution over time [21]. Concept drift is particularly relevant in HR data streams from wearable sensors, where factors such as changes in physical activity, stress, or environmental conditions can change the statistical properties of the data, thus impacting the model’s performance. Figure 2 illustrates the schema employed to generate forecasts from the prepared HR data. The forecasting structure is designed for online processing, treating data as a stream rather than in batches. This is crucial in the context of wearable sensor data, which is constantly updated with new observations. Algorithms that do not adhere to online processing may struggle in real-world production environments, as they rely on historical data that might not reflect current conditions [22]. To address this, we implement two adaptive learning strategies [21].

1. Rolling window. The first strategy involves updating the training data using a rolling window. A fixed size is maintained in the window, which discards the oldest data points while incorporating the most recent ones. By doing so, the model always has access to the latest and most relevant information.
2. Model retraining. Instead of incrementally updating the model parameters, a new model is retrained at each forecasting step. Therefore, the model fully captures the latest data patterns, which may have shifted due to concept drift, rather than relying on previously learned weights. This strategy appears computationally sustainable in the context of short-term HR forecasting and allows the model to better adapt to evolving trends.

To clarify our forecasting strategy, model retraining is conceptually identical to the direct recursive multiple output (DIRMO) strategy [23], although we do not generate multiple outputs simultaneously. Like DIRMO, we retrain the model for each forecasting step, maintaining the model up-to-date.

When dealing with an adaptive learning schema and time series forecasting, researchers should be aware of the type of sliding window they are employing. This parameter can significantly affect the forecast outcome and must be carefully considered [24]. Typically, a sliding window refers to the slice of training data used to make forecasts. In our case, it represents the most recent minutes of HR data, with the number of minutes determined by a parameter we define as the window size. There are two alternative forms of sliding windows: the expanding window and the rolling window [25]. Both methods involve using a dynamic and continuously updating dataset to perform sequential HR forecasts. In an expanding window, there is a fixed origin that retains all past occurrences, whereas a rolling window drops the most distant observations, thus continuously updating the origin. As illustrated in Fig. 2, our approach employs a rolling window with a constant window size. Therefore, at each forecasting step, a fixed slice of the most recent available records is used for prediction. The key feature of the rolling window method is its adaptability to changing data patterns over time by dropping the oldest observations. This ongoing data refreshment ensures that the forecasting model remains up-to-date and responsive to evolving HR trends.

### Test Validation and Experimental Settings

As shown in Fig. 2, out-of-sample (OOS) validation is adopted to ensure that time dependence is respected [26–28]. OOS observations, held out as the test set, follow chronologically after the in-sample observations used for model training. The OOS set should be sufficiently large to ensure reliable findings, with a minimum of 200 observations recommended [26]. Given our 1-min granularity, this corresponds to an evaluation period of over 3 h. In our study, we set the OOS size to 220 min per user. It is important to note that, with a forecast horizon of one, these 220 observations yield 220 one-step-ahead forecasts for evaluating model performance.

A solid foundation of conclusions, in line with the general principle of cross-validation, is primarily inferred by averaging results when coming from numerous instances. To enhance the number of experiments conducted and, hence, the reliability of our validation procedure, multiple experiments are generated from the combination on various dimensions. Variables that are combined to determine unique experimental settings are

- |Users|: data workflow is replicated for each of the participants in the datasets. Subsequently, resulting scores, employed for comparing specification performance, are the averages of the individual scores within this group.
- |Window size|: the choice of this parameter is crucial when dealing with time series forecasting since it affects resulting scores [24]. A set of values for the window size parameter are tested to evaluate its impact. Diverse values allow for a comprehensive assessment, with results presented for each combination of time of the day and window size.
- |Time of the day|: this parameter dictates the timing of data acquisition. It applies only when this information is available and, thus, just for the MMASH dataset. Each user of the MMASH dataset is evaluated during two distinct 3-h periods, one extracted during nighttime and the other during diurnal activities. Tests of different time of the day are presented separately instead of averaging them.

### Formalization of the Validation Structure

A time series is an ordered collection of data points in a time interval, in which each *x*_t_ is an observation at a certain time instant. Let *X*_t_ be a time series of length *t* defined as

1 $$\begin{aligned} X_t = [x_1, x_2, \dots , x_t]. \end{aligned}$$

Forecasts generated during validation could be defined by the vector *X*_N_, where $N$ represents the constant number of OOS observations to predict. However, to better represent the forecasting structure adopted to test our algorithm, we prefer the alternative representation $\hat{X}_{t+1}^{(t+N)}$. In this notation, subscript and superscript identify respectively the first and the last element in the sequence. The vector of forecasts generated at time *t* could be formalized as

2 $$\begin{aligned} \hat{X}_{t+1}^{(t+N)} = [\hat{x}_{(t+1)}, \hat{x}_{(t+2)}, \dots , \hat{x}_{(t+N)}]. \end{aligned}$$

where $\hat{X}_{t+1}^{(t+N)}$ represents the entire vector of forecasts for *N* time steps; $\hat{x}_{(t+1)}$ represents the forecast element generated at time $t+1$, initiating the sequence of forecasts following the present time *t*. The vector $\hat{X}_{t+1}^{(t+N)}$ is estimated as

3 $$\begin{aligned} \hat{X}_{t+1}^{(t+N)} = F_p(X_w) . \end{aligned}$$

where *w* ($w\in [1,W]$) specifies the size of the rolling window at each iteration; *p* refers to the number of iterations necessary to forecast all the *N* OOS observations. Since the *horizon* $H = 1$, *p* corresponds to *N*. Indeed, the number of steps $p = N/H$; $F_p$ represents *p* different forecasting models employed during validation; $X_w$ is a vector containing *w* actual observations which are continuously updated to forecast given the last available values.

### ARIMA Algorithm and the Random Walk Model

ARIMA algorithm is adopted to forecast. ARIMA is a family of models renowned for its ability to capture a wide range of complex temporal patterns. Versatility of the method is a remarkable motivation under its recurrent usage and is primarily attributed to its hyper-parameter tuning: *p*, *d*, and *q*. Its general equation in its explicit form could be expressed as

4 $$\begin{aligned} (1-L)^{d} X_t = \sum _{i=1}^{p}\varphi _i X_{t-i} + \varepsilon _{t} + \sum _{j=1}^{q}\vartheta _j \varepsilon _{t-j} = \dots \quad . \end{aligned}$$

From Eq. 4, components of ARIMA are inspected:

1. Autoregressive (AR) component (*p*): *p* in ARIMA reflects the adaptability of the model to different data processes. It signifies the order of autoregressive terms and, consequently, how far back in time the model reaches to understand data dependencies. A higher *p* accommodates longer-term dependencies, making ARIMA capable of capturing intricate patterns in the data.
2. Differencing (I) component (*d*): *d* in ARIMA represents the order of differencing required to achieve stationarity through the usage of lag operator L. This value caters to different levels of data volatility, ensuring that ARIMA can handle both mildly and heavily trending time series.
3. Moving average (MA) component (*q*): *q* in ARIMA embodies its adaptability to short-term data dynamics. It signifies the order of moving average terms and, consequently, the extent to which ARIMA considers recent noise or errors. A higher *q* allows ARIMA to capture subtle, short-term fluctuations in the data, making it versatile across a spectrum of data complexities.

This adaptability enables ARIMA to capture various data processes. On the one hand, complex time series, presenting intricate and highly volatile data, tend to be better modeled by higher values of *p*, *d*, and *q*. On the other hand, in cases where the data exhibits moderate or mild patterns, a more parsimonious choice of hyper-parameters ensures that model complexity is not needlessly increased, maintaining it particularly effective for capturing simpler processes. A specification refers to a chosen configuration of these hyper-parameters, enabling insight extraction through the assessment of predictive capacity. ARIMA thus emerges as a powerful tool due to its interpretable nature, which facilitates a clear understanding of underlying dynamics.

In this study, addressing our research question involves examining whether simpler models can adequately forecast HR time series. Notably, ARIMA (0,1,0), which corresponds to the random walk model, may serve as a baseline for comparison as it is one of the simplest specification, with only the parameter *d* beyond the constant term. A random walk model employs only two parameters: the intercept and $\mathcal {\sigma }^2$. At each minute, a new forecast is generated by randomly adding a value to the last available observation, captured by the parameter intercept. The magnitude of this added value is regulated by the $\mathcal {\sigma }^2$ parameter, which captures the recent variance of the HR process.

### Residual Diagnostics

Residual diagnostics represents a promising tool, sometimes overlooked in machine learning research which focuses on comparative analysis for assessing predictive performance. Box and Pierce [29] suggested that, after identifying the best specification, residuals should be analyzed to evaluate whether the model still lacks of fit. The Shapiro-Wilk and Kolmogorov-Smirnov tests are adopted to evaluate the normality of residuals [30]. To assess the independence of residuals, autocorrelation is examined, as its presence may suggest that the algorithm is not capturing all explainable variance. The Durbin-Watson test [31] generates a test statistics where values outside the interval (1.5, 2.5) indicate presence of autocorrelation in the residuals. Traditionally, diagnostic methods are used to assess model fit, with normality and independence in residuals being desirable properties. However, in this study, instead of relying solely on statistical tests, we prioritize visual inspection to gain deeper insights into the random walk model. We deliberately opted for the histogram distribution due to the practical difficulty of plotting multiple residual plots simultaneously with the other techniques. Therefore, we examine the distributional patterns of the forecasts generated, focusing on studying the shapes approximating residual distribution. This approach combining visual and statistical diagnostics supports our investigation into the random walk model.

### Evaluation Metrics

The selection of a particular evaluation metric depends mostly on the interpretability and comparability with prior studies. Typically, two of the most widely used evaluation metrics are mean absolute error (MAE) and mean absolute percentage error (MAPE). MAPE, expressed in percentage, offers an intuitively straightforward measure of accuracy. However, its adoption requires caution due to its susceptibility to generating misleading metrics when dealing with actual values ranging in the interval (-1, 1). Indeed, inflated percentage errors arise from the use of these values as denominators. In HR forecasting, this limitation may occur especially if other researchers apply differentiation before processing data. As such, MAPE should be avoided in this context, with MAE representing a more optimal alternative. MAE strength relies on its simplicity, as it reports errors on the same scale or in the same units as the original data. We argued that MAE is preferred over its relative transformation (i.e., root mean square error or mean square error), given the absence of evidence in the literature to support the attribution of different weights in this scenario.

## Experiments

Autoregressive methods have demonstrated superior performance when addressing HR forecasting issue. However, researchers have not inspected which is the optimal specification of ARIMA. Therefore, a comprehensive forecasting analysis is conducted by comparing performance of various specifications of ARIMA hyper-parameters. We aim at generating an interpretable model in which hyper-parameter combination may provide crucial insights.

### Experimental Setup

To assess which is the best specification of ARIMA in a minute-by-minute forecast, different combinations are tested. Specifically, a reduced set of candidates has been identified by comparing performance in a step-wise manner. In the optimization process, first tests suggested that the algorithm tends to exhibit inferior performance when specifications contain larger values. Therefore, the following conditions are defined to identify the set of specifications to be tested: *(i)* *p* lower than 5; *(ii)* *d* lower than 2; *(iii)* *q* lower than 5. However, some exceptions with larger values are included in the results to provide insights that reinforce the tendencies mentioned above.

Window sizes are studied by comparing the performance of the algorithm on a limited set of values. The algorithm is tested on the following values: 15 min, 30 min, 45 min, 90 min, and 150 min. Instead of just selecting the optimal value, resulting scores are displayed for each one of the window size value to enable an assessment of algorithm behavior. Tests are not only generated separately with respect to the window sizes, but also for each dataset. Furthermore, with regard to the MMASH dataset, tests are duplicated according to the parameter time of the day, which regulates the selection of the slice of data. The distinction between day and nighttime data is necessary since HR properties differ significantly, with higher BPM values typically observed during daytime activities. In our case, MAE, a scale-dependent measure, would have captured more bias from tests performed during the day, averaging larger numerical values [27].

**Fig. 3:**
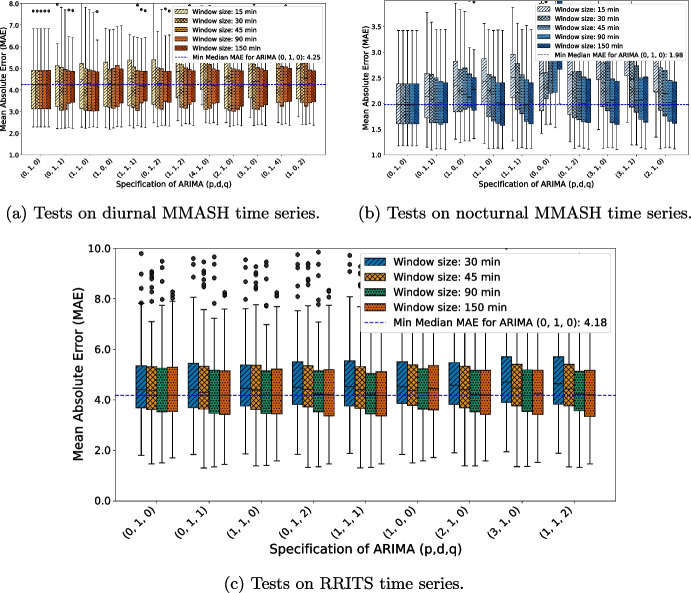
Each boxplot displays the mean absolute error (MAE) for the top six performing ARIMA specifications. The first two plots present MAE results from the MMASH dataset, with the left plot (in warm colors) for the diurnal time series and the right plot (in cool colors) for the nocturnal time series. Below these, the boxplot from the RRITS dataset is shown. In all figures, each specification is assessed over different window sizes, with the resulting MAE displayed accordingly. A horizontal dashed line indicates the lowest median MAE across all window sizes and specifications, with the relative minimum median value noted in the legend

### Forecasting Results of Most Performing Specifications

Figure 3 presents the boxplots of MAE scores derived from the time series of the MMASH dataset, showing separate figures for each time period (diurnal and nocturnal), with the RRITS dataset plotted below. Boxplots are used here to analyze the distribution of MAE scores, where each data point corresponds to the average error for an individual user under a specific experimental condition. Therefore, this illustration offers a comprehensive view of variations in the error metric across each setting. Instead of selecting only the top-performing specification, we opted to display the top six performing ARIMA specifications based on their median performance (indicated by the horizontal line within each box). The selection was applied before plotting by separately assessing MAE scores for each dataset and for each window size. Consequently, figures display more than six specifications if the most performing models varied across window sizes. For example, the figure on nocturnal time series for MMASH includes 10 distinct specifications, which differ in part from those selected for the diurnal time series. The following observations can be drawn from the figure:

- Limited exploitation of past information by ARIMA specifications. In the top six performing, the most performing are the simplest, indicating limited reliance on historical data in univariate HR forecasting. Specifically, ARIMA (0,1,0), a random walk model, ranks as a top performer. Similar weak models, such as ARIMA (1,1,1) and (0,1,1), yield comparable results. This trend suggests that little information needs to be leveraged from the inclusion of past values or error terms for improving forecasting accuracy.
- Assessment of window sizes performance. It seems that 90-min and 150-min window sizes perform better overall. Particularly, more complex specifications benefit more from a wider window size as they need more data to train on. Indeed, the simplest model, the random walk, exhibits the same score across all the different window sizes in the MMASH dataset, while slightly differs just in the RRITS dataset. However, while the benefits of larger window sizes are more evident for complex models, as yet stated, forecasting does not need complex models, even if trained on a wide window size.
- Necessity of differentiation. It seems that including one term of differencing, *d* equal to one, generally enhances forecast accuracy.
- Comparison of diurnal, nocturnal, and RRITS MAE. Diurnal data from MMASH generally result in higher MAE compared to nocturnal data, yet this does not necessarily indicate poorer performance during the day since MAE is scale-dependent, and diurnal BPM tends to be higher. Additionally, the RRITS results closely mirror the MMASH diurnal findings, both in MAE and in the model specifications. This similarity suggests that RRITS may capture daytime activity and exhibit similar HR variability patterns to MMASH diurnal data.

**Fig. 4:**
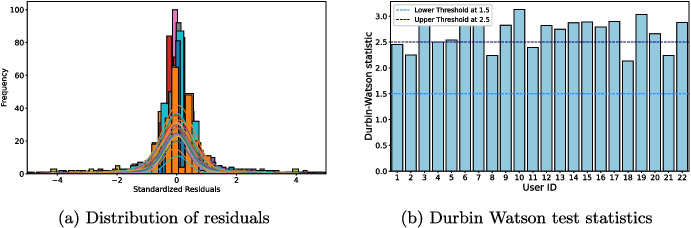
Residual diagnostics of the 22 users in the MMASH dataset. Normality is evaluated from the distribution of residuals. In the y-axis is reported the frequency, whereas in the x-axis is the value of standardized residuals. Durbin Watson statistics are employed to assess the presence of autocorrelation in residuals. Values above the threshold at 2.5 are associated to negative autocorrelation, while below threshold at 1.5 to a positive autocorrelation

### Random Walk Inspection Through Residual Analysis

Additional insights into forecasting model performance could be elicited from analyzing residuals. Residuals of the random walk model from the 22 MMASH user time series are inspected. *P*-values from the Shapiro Wilkinson and Kolmogorov-Smirnov tests, collected in Table A1, do not support the statistical normality of residuals. However, for our study, it is more relevant to examine graphically the residuals in detail to better assess the random walk nature and adequacy. Figure 4 displays histograms of standardized residuals. Even if most curves in the figure roughly approximate a normal distribution, the standard deviations appear lower than that of a standard normal distribution. Unlike a standard normal distribution where about 68% of data lies within ± 1 interval, less variability is evident in the nearly zero, flattened tails. Therefore, residuals are predominantly concentrated around 0, which could be associated to a good performance of the predictor. Lastly, the Durbin Watson test is performed to assess whether autocorrelation is present in the residuals. The test statistics, collected in Table A1 and displayed in Fig. 4, are predominantly near the upper threshold of 2.5, indicating a tendency toward negative autocorrelation.

**Fig. 5:**
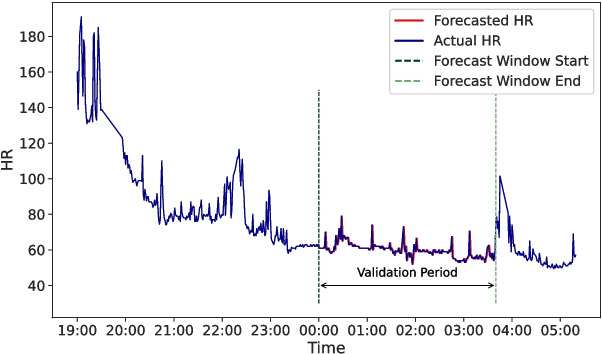
Forecasts of the random walk model (red) against actual values (blue). Forecasts of one random user are extracted and plotted against the relative actual values to visually inspect the goodness of the prediction. On x-axis, time expressed in hours indicates that forecasts are those generated during nighttime. ARIMA (0,1,0) is employed to carry out these forecasts from a time series of the MMASH dataset

## Discussion

In Fig. 5, forecast HR values initially appear to align closely with actual HR values. However, upon closer inspection, the forecast sequence behaves more like a shifted version of the actual sequence. This observation raises a key question about the role of historical HR data in informing future predictions within a univariate and short-term forecasting setting. By evaluating different ARIMA specifications, we aim to determine how past data is leveraged to generate forecasts.

In finance, random walk theory posits that market prices follow an unpredictable path, making historical information minimally informative for future predictions [11]. As Fama noted, the random walk model may serve as a baseline for assessing analyst performance [11]. Similarly, we use ARIMA (0,1,0), which corresponds to the random walk model, as a benchmark to address our research question.

Our findings indicate that, despite the recent trend of using complex machine learning and deep learning models, the random walk model remains competitive, if not superior, as a forecasting baseline. This outcome may be surprising, given that the random walk model only captures stochastic variance around recent values by regulating a single parameter beyond the intercept. For this reason, one might expect the random walk model to often fall short. However, research in financial forecasting has explored why even sophisticated models struggle to outperform the random walk model [32], emphasizing that this challenge is not uncommon. Furthermore, residual diagnostics support this observation by providing insights into the forecast accuracy of the random walk model. While non-normality in residuals is traditionally viewed as a potential limitation, this does not necessarily represent a drawback [33]. In this case, forecast accuracy seems to be acceptable, as a high concentration of residuals around zero corresponds to effective predictor performance. Additionally, the tendency of negative autocorrelation aligns with the random walk process, where values shift randomly from the previous observation.

## Related Works

The advent of wearable technologies in recent years has fostered further research on HR time series. In the literature, there is a plethora of studies analyzing HR, with several research directions. For instance, some researchers explore the inferential implication of HR variability, particularly its relevance to the activities of the nervous system [34–36]. Another significant research area is the development of early warning systems for detecting cardiac conditions, which is primarily led by ECG-based methods. Today, ECG information can be continuously gathered by wearable sensors. The time intervals between two consecutive heart beats, which we use in our analysis, are embedded within the ECG waves, specifically measuring the elapsing time between two R peaks. This is underscored in our second dataset, “RR interval time series from healthy subjects” (RRITS), which employs a Holter monitoring device to collect ECG data. Most efforts in this domain focus on detecting the presence of arrhythmia, though recent research has shifted towards screening for arrhythmias onset in advance [37, 38].

Although findings from ECG-based studies are promising and often supported by comprehensive methodologies, the reliability of many other current early detection systems remains questionable due to their reliance on cross-sectional data [39–41]. These attempts to develop early detection systems typically use RR intervals and other physiological parameters, without leveraging full ECG information. The Cleveland dataset or similar surrogates are often used, which only offer a snapshot of clinical conditions. We argue that findings based on such datasets are misleading, as they fail to capture the longitudinal nature of cardiac events, making it challenging to claim that reliable alarm systems can be derived from this data. To our knowledge, the few studies that have developed proper real-time monitoring systems without ECG data focus on specific event onsets, such as heart failure or neonatal sepsis [9, 10].

Finally, we turn our attention to the studies most closely related to our work, which focus on forecasting HR in a univariate fashion to develop detection systems. These studies operate under the assumption that accurate forecasting could enable early detection of pathological conditions. This fosters us to assess the predictive power of models, to determine whether this approach is a viable path forward. Furthermore, it has been highlighted that many machine learning practitioners may not be fully aware of specific guidelines related to time series forecasting [24, 27, 28, 42]. Failure to adhere to these guidelines can compromise the validity of findings. Moreover, insufficiently detailed descriptions of the methodology may hinder meaningful comparative analysis among different studies [27]. When reviewing the methodologies used in these studies, it seems that these issues persist, especially when implemented in an online fashion. For example, Luo and Wu [43] do not mention the use of sliding windows and horizons, a non-trivial shortcoming. Conversely, key details (e.g., window size, the horizons, and data acquisition timing) are provided by Lin et al. [3]. However, it is not explicitly clarified whether ARIMA model weights are retrained at each step. Additionally, it is merely tested a particular specification, ARIMA (2,0,0). Four different deep learning models are compared using simulated HR data from the MIMIC database by Alharbi et al. [4]. The forecasting process is clearly outlined, including the lengths of sliding window and horizons. However, the pre-processing steps involve unnecessary transformations of HR time series. Specifically, statistical standardization is applied, which is typically employed only in multidimensional analysis before clustering or distance-based methods. Moreover, just one singular time series of a patient has been extracted, making questionable the validity of the findings. To forecast HR values, we employ autoregressive models. It is important to note that while machine learning and deep learning models have been explored in other works, they have not shown significant improvements over ARIMA in the univariate case. An extensive list of the most important machine learning as well as deep learning models have been proved to underperform compared to ARIMA [5]. Moreover, long-short term memory (LSTM) performances have been tested, and compared to a simple AR (3) model which fits better than power neural networks, AR is a specific case of ARIMA [6]. The performance of autoregressive models against deep learning ones is tested through a comparative analysis by Staffini et al. [6], specifically involving LSTM networks. The study is designed to generate forecasts as minute-based streams, employing an extended 10-day study to provide a robust foundation. The study just lacks details about the adaptive strategies (e.g., sliding window) which are almost surely implemented but not mentioned. Generally, the granularity in these forecasting works is set to 1 min, offering several benefits as outlined in Section [2.2]. Presumably, a granularity at the inter-beat level, on average less than 1 s, is adopted by Oyeleye et al. [5]. However, this choice hinders comparability with other works using a minute-scale granularity. Forecasting at the inter-beat level typically results in much lower error rates compared to minute-based forecasts. Moreover, predicting values every second may render the solution unsustainable for the deployment.

When dealing with residual diagnostics, various methods can be employed to assess the normality and independence of residuals. The most renowned graphical techniques include the normal Q-Q plot, the normal P-P plot, residuals vs. fitted values plot, autocorrelation function (ACF) plot, and the histogram distribution [44]. Each of these methods offers unique insights:

- Normal Q-Q plot: this plot compares the quantiles of the residuals with the quantiles of a normal distribution, helping to visually assess deviations from normality.
- Normal P-P plot: similar to the Q-Q plot, the P-P plot compares the cumulative distribution function of the residuals with the expected cumulative distribution of a normal distribution.
- Histogram distribution: this method visualizes the frequency distribution of the residuals and can be used to detect skewness, kurtosis, and other departures from normality.
- Residuals vs. fitted values plot: this plot helps in detecting non-linearity, unequal error variances, and outliers. Residuals should ideally be randomly dispersed around the horizontal axis.
- Autocorrelation function (ACF) plot: autocorrelation of the residuals is displayed at different lags, helping to detect any patterns suggesting non-independence.

## Conclusion

This study presents a comprehensive evaluation of ARIMA-based univariate methods for real-time, minute-by-minute HR forecasting, designed to align with prevailing state-of-the-art practices. Our findings reveal that the random walk model performs competitively, underscoring a critical insight: refining model complexity offers minimal benefit in univariate and short-term forecasting. This suggests that, in this context, historical HR data alone may lack the informational depth needed for significant predictive improvements.

In light of these findings, it becomes evident that the current focus on comparative analysis with increasingly complex models may be misdirected. The limitation lies not within the algorithms themselves, but within the constraints of the univariate, short-term methodology. As such, meaningful advancements may instead be realized by expanding this approach to incorporate multivariate data, such as additional physiological measures, or by focusing on alternative metrics like HR variability.

Moreover, finance research suggests that the random walk model strong performance in short-term horizons may be due to the limited need for historical data beyond recent observations [32]. In short-term forecasting, the need for additional historical data may be minimal, favoring models that rely primarily on recent observations and stochastic variation. Therefore, a shift toward longer forecasting horizons, even if reducing forecast accuracy, could reveal additional patterns over extended timeframes. However, this approach would necessitate larger datasets, as the current 24-h length may be insufficient to support training for longer-term patterns effectively.

In summary, pursuing these alternative directions could aid in developing real-time alarm systems capable of meeting clinical demands for timely and reliable cardiovascular monitoring.

## Supplementary information

Tables of MAE scores for all users, across each dataset, and window size are also available in our private GitHub repository. For review purposes, a zip file containing these tables has been included in the Supplementary Material.

## Supplementary Information

Below is the link to the electronic supplementary material.

Supplementary file 1 (zip 93 KB)

## Data Availability

The dataset MMASH employed in this study has open access on PhysioNet under the Open Data Commons Open Database v1.0 license at the following web URLs: https://physionet.org/content/mmash/1.0.0/, whereas the dataset RRITS has open access on PhysioNet under the Creative Commons Attribution 4.0 International license at the following web URLs: https://physionet.org/content/rr-interval-healthy-subjects/1.0.0/ .

## Appendix A. Test Statistics for Normality and Independence of Residuals

**Table 1:** Test statistics for the different 22 users

User ID	Shapiro *p*-value	Kolmogorov *p*-value	Durbin Watson statistic
1	0.0001	0.0001	2.4551
2	0.0001	0.0001	2.2509
3	0.0001	0.0001	2.9562
4	0.0001	0.0001	2.5004
5	0.0001	0.0001	2.5385
6	0.0001	0.0001	2.9281
7	0.0001	0.0001	3.0163
8	0.0001	0.0001	2.2408
9	0.0001	0.0001	2.8275
10	0.0001	0.0001	3.1298
11	0.0001	0.0001	2.3966
12	0.0001	0.0001	2.8229
13	0.0001	0.0001	2.7487
14	0.0001	0.0001	2.8758
15	0.0001	0.0001	2.8884
16	0.0001	0.0001	2.7918
17	0.0001	0.0001	2.9005
18	0.0001	0.0001	2.1311
19	0.0001	0.0001	3.0330
20	0.0001	0.0001	2.6592
21	0.0001	0.0001	2.2380
22	0.0001	0.0001	2.8799

Shapiro and Kolmogorov are tests for normality, while Durbin Watson statistics are used to assess autocorrelation of residuals
